# Real-Time Myocardial Infarction Detection Approaches with a Microcontroller-Based Edge-AI Device

**DOI:** 10.3390/s24030828

**Published:** 2024-01-26

**Authors:** Maria Gragnaniello, Alessandro Borghese, Vincenzo Romano Marrazzo, Luca Maresca, Giovanni Breglio, Andrea Irace, Michele Riccio

**Affiliations:** Department of Electrical Engineering and Information Technology (DIETI), University of Naples Federico II, 80125 Naples, Italy; maria.gragnaniello@unina.it (M.G.); alessandro.borghese@unina.it (A.B.); vincenzoromano.marrazzo@unina.it (V.R.M.); luca.maresca@unina.it (L.M.); giovanni.breglio@unina.it (G.B.); andrea.irace@unina.it (A.I.)

**Keywords:** deep learning, edge computing, machine learning, myocardial infarction detection

## Abstract

Myocardial Infarction (MI), commonly known as heart attack, is a cardiac condition characterized by damage to a portion of the heart, specifically the myocardium, due to the disruption of blood flow. Given its recurring and often asymptomatic nature, there is the need for continuous monitoring using wearable devices. This paper proposes a single-microcontroller-based system designed for the automatic detection of MI based on the Edge Computing paradigm. Two solutions for MI detection are evaluated, based on Machine Learning (ML) and Deep Learning (DL) techniques. The developed algorithms are based on two different approaches currently available in the literature, and they are optimized for deployment on low-resource hardware. A feasibility assessment of their implementation on a single 32-bit microcontroller with an ARM Cortex-M4 core was examined, and a comparison in terms of accuracy, inference time, and memory usage was detailed. For ML techniques, significant data processing for feature extraction, coupled with a simpler Neural Network (NN) is involved. On the other hand, the second method, based on DL, employs a Spectrogram Analysis for feature extraction and a Convolutional Neural Network (CNN) with a longer inference time and higher memory utilization. Both methods employ the same low power hardware reaching an accuracy of 89.40% and 94.76%, respectively. The final prototype is an energy-efficient system capable of real-time detection of MI without the need to connect to remote servers or the cloud. All processing is performed at the edge, enabling NN inference on the same microcontroller.

## 1. Introduction

Myocardial infarction (MI) is pathologically defined as the death of myocardial cells resulting from prolonged ischemia [1]. The expansion of MI is quite fast, and, if not diagnosed and treated promptly, it continues to damage the myocardial structure and the left ventricle’s function [2]. According to statistics from the World Health Organization, among various forms of cardiovascular diseases, MI has the highest mortality rate [3]. Sometimes, MI can initially occur silently. Silent Myocardial Infarction happens without chest pain or other noticeable symptoms, often leaving patients unaware of their condition [4]. Monitoring methods outside of hospital facilities and the development of automatic MI detection have become lifesavers.

The evaluation of alterations in cardiac electrical activity using a 12-lead ECG is a common practice for diagnosing and assessing the risk of MI. Specifically, the 12 leads can be used to specify the region of myocardial damage. Gupta et al. quantified the contributions of each lead of the ECG signal from the Physikalisch-Technische Bundesanstalt (PTB) diagnostic database [5] and observed that it is possible to identify MI upon processing only ten seconds of raw ECG data from the V6, Vz, and II leads [6]. This finding holds significance in the context of wearable devices for everyday health monitoring, where acquiring a single-lead ECG is feasible (e.g., chest straps, smartwatches, or an ECG patch like SmartCardia [7]). It is also anticipated that many more devices will acquire the capability to capture ECG signals. The use of the II-lead ECG for real-time MI detection is appropriate in these scenarios, aligning with the need for efficient and accessible health monitoring solutions.

A large body of literature focuses on the methods for ECG signal analysis to detect and predict MI [8,9,10]. Early methodologies were primarily centered around the analysis of morphological features within the ECG signal. These features included ST-segment deviations and alterations in the shapes of T and Q waves [11]. These approaches, including Fast Wavelet Transform to Continuous Wavelet Transform and Fourier Intrinsic Band Functions (FIBFs), found utility in processing ECG data for ischemia and MI detection [12,13,14]. Vectorcardiography has emerged as a promising technique, driving numerous studies to detect ischemic beats and diagnose MI. Spectrograms, capturing frequency variations over time in ECG signals, have proven valuable for training and classifying CNNs [15]. A variety of classifiers were applied, including NNs, Support Vector Machines (SVMs), K-Nearest Neighbors (k-NNs), decision trees, and Random Forests (RFs) [16,17,18,19]. Recent trends indicate a shift from traditional ML techniques to DL methods for MI detection [10,20].

While these methods have demonstrated exceptional outcomes, their computational complexity often makes them incompatible with limited-resource hardware, impeding real-time processing. Currently, this challenge is addressed through a two-step strategy. A wearable system (e.g., chest straps and smartwatches) is designed to capture physiological signals (e.g., ECGs and PPGs) and transmit them via short-distance protocols, such as Bluetooth, to a mobile phone. The latter subsequently sends the data to the cloud for processing. However, this approach is limited by the diminishing of the battery life of the wearable device due to the continuous transmission of a substantial amount of raw data to the cloud. Moreover, it introduces latency, impeding real-time monitoring and detection in healthcare applications.

In this context, Edge Computing presents an efficient solution to mitigate computational complexity and consumption. With this innovative paradigm, all processing occurs on a single device (i.e., on the wearable device itself), and only the classification results are transmitted to the cloud for remote monitoring. This approach addresses the aforementioned issues related to energy consumption, latency, and privacy vulnerabilities.

This study delved into the design, development, and validation of an energy-efficient MI detection system, custom-built for low-power wearable devices, with a particular emphasis on a single-lead ECG, the II-lead one. The distinctive features of this research included: (i) the use of the Edge Computing paradigm, which differed from traditional IoT approaches by enabling offline data processing, alleviating the burden on the internet infrastructure. (ii) The evaluation of two distinct methods, highlighting the contrasting capabilities of ML and DL. The first method was rooted in Fourier Intrinsic Band Functions (FIBFs) [14], while the second method employed Spectrograms. Both methodologies were performed on the same microcontroller and experimentally verified to assess their feasibility in real-time.

The final prototype could process acquired ECG signals in real-time through Edge Computing algorithms implemented on the same microcontroller responsible for signal acquisition and processing result display.

The remaining Sections of this paper are structured as follows: Section 2 critically reviews the current literature within the field. Section 3 is dedicated to presenting the architecture of the prototype and the two proposed methods. It provides a detailed description of the data preprocessing and NN training phases for both methods. Section 4 presents and discusses the training results in terms of accuracy, processing time, and memory usage. Finally, Section 5 summarizes the study and collects the main findings.

## 2. Related Works

In this Section, a collection of studies documented in the literature is described. It is worth noting that some of these studies utilized the 11-lead ECG, while others, like the one presented in this article, employed the II-lead one.

The authors in [21] proposed a prototype for a real-time heart attack detection and warning system designed to mitigate road accidents. However, its implementation necessitated the use of two subsystems: a wearable sensor subsystem and an intelligent heart attack detector. The first was responsible for signal acquisition and transmission to the latter subsystem, which conducted classification and issued alarms, when necessary, also sharing the driver’s location. Nevertheless, this system fell short of being entirely wearable, given its composition of interconnected systems, inevitably resulting in high power consumption.

In [22], researchers proposed a real-time event-driven classification technique for MI detection using the SVM algorithm with a two-level classifier. They applied denoising through a 32nd-order zero-phase bandpass filter and segmentation for a 47-feature extraction. The method demonstrated an accuracy of 90% when evaluated on a single-lead ECG from the PTB database. The authors in [23] opted for a five-level RF classifier featuring 100 decision trees extracting 72 features. The achieved results demonstrated an accuracy of 80.32%. While [22,23] aimed to streamline classifier complexity for energy efficiency, they heavily relied on extensive feature engineering to identify the optimal features. Moreover, the resource-intensive nature of the feature extraction process in terms of time and energy rendered these methods incompatible for real-time MI detection on wearable devices [24].

In [25], researchers employed the k-NN algorithm for the classification of normal and MI heartbeats, utilizing an 11-lead ECG from the PTB database. The R-peaks were identified using the Pan–Tompkins algorithm [26]. The segmentation of each portion, spanning 651 samples, involved the application of a four-level Discrete Wavelet Transform (DWT) using db6. Subsequently, 47 features were extracted, serving as inputs for the k-NN classifier to categorize each ECG segment. The classification accuracy achieved was notably high, reaching 98.80%. Nevertheless, this method demanded a high level of computational complexity, rendering it unsuitable for wearable devices with constrained resources but applicable in clinical setups. In [27], researchers employed a DL algorithm using a CNN. Their approach adopted 1D deep CNN architecture comprising four convolutional layers, four max-pooling layers, and three fully connected layers. When applied to II-lead ECG data from the PTB database, their method demonstrated an accuracy of 95.22%. However, as in [25], the viability of their approach was limited to clinical setups. In [28], the Binary Convolutional Neural Network (BCNN) emerged as an innovative solution. This model efficiently addressed the time-consuming feature extraction process associated with convolution and exhibited a reduced memory requirement. The initial phase involved preprocessing raw ECG data, incorporating denoising through a bandpass filter, and segmenting based on R-peak detection. To further optimize memory usage, a strategic reorganization of CNN layers was implemented, drawing inspiration from the embedded Binarized Neural Network (eBNN) technique. In the eBNN approach, the convolution result undergoes a direct transition to pooling, followed by batch normalization and binary activation, thereby eliminating the necessity for intermediate storage. The authors in [24] introduced an innovative Template-Matching-based Early Exit (TMEX) CNN architecture that enhanced the energy efficiency of the baseline architecture while maintaining comparable performance. The evaluation of their methodology was conducted on two datasets, the PTB and PTB-XL [29]. Using the PTB dataset and 11-lead ECG, they achieved an accuracy of 99.24%. Conversely, with the II-lead ECG of the PTB-XL, the results were an accuracy of 84.24%. They conducted a memory usage study using the EFM32 Giant Gecko Development Board, and it showed a utilization of 20 kB. In [30], a multilayer Long Short-Term Memory (LSTM) NN was developed, incorporating a preprocessing pipeline for real-time identification of infarcted patients from a single heartbeat of a single-lead ECG. The process was designed to be frequency-independent within a specified and targeted range of sampling frequencies. The II-lead ECG signal undergoes filtration through a 45 Hz low-pass filter and a 500 ms moving average filter, followed by the R-Spike detector, that continuously provides the locations around which heartbeats are segmented. These samples are then forwarded to a classifier that comprises four layers exclusively composed of LSTM units.

## 3. Materials and Methods

### 3.1. System Prototype

**Figure 1:**
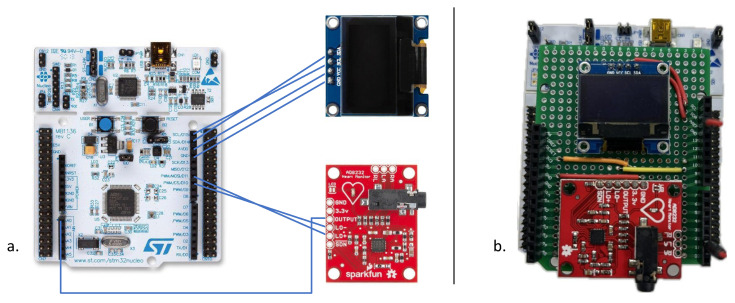
(**a**) System architecture of the prototype; (**b**) final prototype showing the AFE to sense the ECG signal and the OLED display for viewing the classification result placed on a shield.

The prototype architecture, designed and developed as depicted in Figure 1, consisted of three fundamental parts: a microcontroller board, NUCLEO-F401RE [31], manufactured by STMicroelectronics, an analog front-end (AFE) to sense the ECG (AD8232) [32], and a display for viewing the classification result (OLED SSD1306) [33].

The adopted development board was a 32-bit microcontroller (MCU) with an ARM Cortex M4 [34] processor operating at a clock frequency of 84 MHz. It was equipped with 512 kB of Flash memory and 96 kB of RAM. It featured a 12-bit Analog-to-Digital Converter (ADC) for acquiring analog signals and two general purpose Direct Memory Access (DMA) controllers, each with 8 channels.

An SSD1306 OLED display is a type of dot matrix graphical screen that utilizes Organic Light-Emitting Diode (OLED) technology. It has a significantly lower power consumption compared to other displays, making it suitable for energy-efficient applications. It boasts a maximum resolution of 128 × 64 pixels and utilizes $I^{2}C\;$ serial protocol.

**Figure 2:**
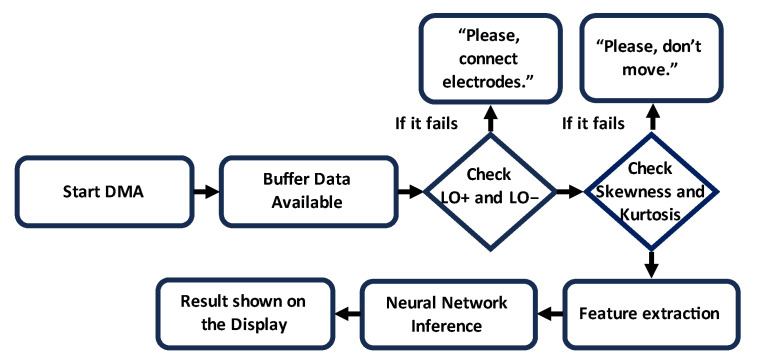
Schematic flowchart for detection of MI using NUCLEO-F401RE.

An AD8232 is an integrated chip serving as an Analog Front End (AFE) designed for the detection, amplification, and filtering of an ECG lead. It provided a common-mode rejection of 80 dB, covering disturbances in the range of 0 to 60 Hz. The input signal was amplified with a gain of 100. The development board had Leads-Off Comparator Output pins, which gave information about the electrode position. During the signal acquisition, Skewness and Kurtosis were constantly monitored to ensure the validity of the data. Figure 2 summarizes the basic steps involved in the developed algorithm.

### 3.2. Database and Built Dataset

**Figure 3:**
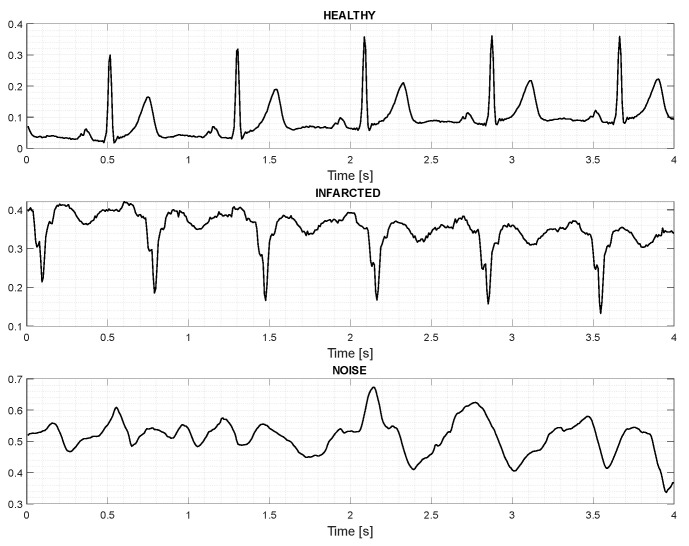
ECG signals for each label used for the second method of NN training.

The PTB diagnostic ECG database [5] and CEBSDB database [35] were used. Furthermore, using MATLAB R2023b, noisy signals were generated to expand the built dataset. Some healthy ECGs from D1NAMO ECG Glucose Data [36] were used to validate the system. The built dataset comprised 612 signals with a split of 80/20% between training and testing data. From these databases, several processing steps were performed to make it compatible with the available hardware. In Figure 3, from the built dataset, an example of an ECG signal lasting 4 s for each label is shown.

**Figure 4:**
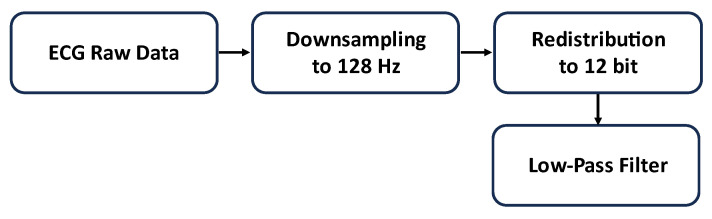
Block diagram of the preprocessing steps.

The ECG signals underwent down-sampling to 128 Hz and were subsequently processed to redistribute the samples across the 12 bits, a necessary adjustment for the onboard ADC. Additionally, prior to utilizing the Analog Front End (AFE), a low-pass filter with a cutoff frequency of 60 Hz was integrated as shown in Figure 4.

### 3.3. Method 1: Fourier Intrinsic Band Functions (FIBFs)

**Figure 5:**
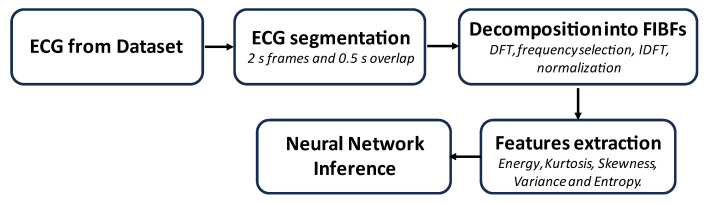
Block diagram of the first MI detection algorithm proposed.

The first method was based on the approach proposed in [14]. Once the pre-processed ECG was obtained (i.e., down-sampled, redistributed, and filtered), the processing involved the following sequence of operations, as described in the block diagram in Figure 5.

#### 3.3.1. Method 1: Data Processing and Feature Extraction

The ECG was segmented into 2 s frames with a 0.5 s overlap, and for each frame, decomposition into bands, known as FIBFs, was performed using the Fourier Decomposition Method (FDM). Each FIBF retained information about the corresponding frequency content to characterize the dynamic behavior of the heart [37,38].

First, to decompose a signal (e.g., $x\left[n\right]$) into a set of desired frequency bands, the signal was modeled as:

(1) $$x\left[n\right]=c_{0}+\sum_{i=1}^{M}y_{i}\left[n\right]=c_{0}+\sum_{i=1}^{M}c_{i}\left[n\right],$$

where $c_{0\;}$ is the mean value of signal $x\left[n\right]$, and ${\left\{y_{i}\left[n\right]\right\}}_{i=1}^{M}$ and ${\left\{c_{i}\left[n\right]\right\}}_{i=1}^{M}$ are the M orthogonal and LINOEP (Linearly Independent Non-Orthogonal Expansion Projector) components, respectively.

Second, the Discrete Fourier Transform (DFT) of the signal $x\left[n\right]$ was obtained, and then a bank of zero-phase filters was applied to select the frequency content of each band:

(2) $$X[k]=\frac{1}{N}\sum_{n=0}^{N-1}x\left[n\right]\;e^{-\frac{j2\pi kn}{N}}\;\;\;\;$$

(3) $$H_{i}\left[k\right]=\left\{\begin{array}{ll} 1, & \;\;(K_{i-1}+1)\leq k\leq K_{i}\;{\;\;\&\;\;\;(N-K}_{i})\leq k\leq {(N-K}_{i-1}-1)\\ 0, & \;\;otherwise \end{array}\;\;\;\;\;\;\;\right.$$

Third, by using the inverse DFT (IDFT) on each of the selected four frequency signals, the FIBFs were computed. The i-th FIBF will be:

(4) $$y_{i}\left[n\right]=\sum_{k=0}^{N-1}\left[H_{i}\left[k\right]\;X\left[k\right]e^{\frac{j2\pi kn}{N}}\right]$$

To enhance the robustness of the AI algorithm, each FIBF was normalized, and a set of features was calculated for each FIBF, including Energy, Kurtosis, Skewness, Variance, and Entropy using the following expressions:

(5) $$Energy=\sum_{n=0}^{N-1}{\left|y_{i}\left[n\right]\right|}^{2}$$

(6) $$Kurtosis=\frac{\frac{1}{N}\sum_{i=0}^{N-1}{\left(y_{i}-\bar{y}\right)}^{4}}{\sigma^{4}}$$

(7) $$Skewness=\frac{\frac{1}{N}\sum_{i=0}^{N-1}{\left(y_{i}-\bar{y}\right)}^{3}}{\sigma^{3}}$$

(8) $$Variance=\frac{1}{N}\sum_{i=0}^{N-1}{\left(y_{i}-\bar{y}\right)}^{2}$$

(9) $$Entropy=-\sum P(n)\;*\;{log}_{2}P(n)$$

where $P\left(n\right)$ is the probability distribution computed as:

(10) $$P\left(n\right)=\frac{S(n)}{\sum S_{i}},$$

and $S\left(n\right)\;$is the squared magnitude of the FFT.

In the end, for improved compliance with a microcontroller-based embedded application, as opposed to the recommended 4 s [14], a 2 s observation interval was selected for the ECG, providing adequate coverage for at least one complete P-QRS-T cycle, representing a full heartbeat. Also, each frame was optimized to include 256 samples, as opposed to 4096. An additional optimization involved the utilization of 4 FIBFs instead of 6. However, the use of overlapping windows provided the NN with a greater number of features to work on.

#### 3.3.2. Method 1: Neural Network Design and Training

The Neural Network, serving as a classifier, was designed and trained using the Edge Impulse development platform [39] and subsequently deployed on the selected MCU. The training process involved 250 epochs with a learning rate of ${8\times 10}^{-4}$. Table 1 provides a summary of the parameters used in the final training.

The NN was fully connected, characterized by two dense layers, each composed of 128 and 80 neurons, respectively. To avoid overfitting, a dropout technique was employed. The architecture is shown in Table 2.

### 3.4. Method 2: Spectrogram Analysis

**Figure 6:**
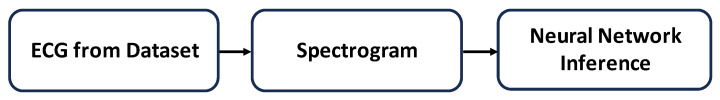
Block diagram of the second proposed MI detection algorithm.

One of the most common methods for highlighting ECG features is through Spectrogram processing. As depicted in Figure 6, this method was relatively straightforward during the processing phase but required a more complex NN.

#### 3.4.1. Method 2: Data Processing and Feature Extraction

A Spectrogram visually represented the spectrum of frequencies over time, providing a graphical representation of both the frequency and amplitude of each component. This representation was crucial for capturing essential information embedded in the ECG signal. This method, typically of a DL approach, achieved an accuracy greater than the ML approach described in Section 3.3. To generate a Spectrogram, the signal was divided into frames of 1.4 s, and an FFT based on 128 samples was calculated for each of them. Table 3 summarizes the set of Spectrogram parameters.

#### 3.4.2. Method 2: Neural Network Design and Training

Although this method also served as a classifier, it necessitated a more complex NN compared to the previous one. Table 4 summarizes the parameters employed in the final training of the second investigated method.

The NN took 1495 features as inputs, which were all the components of the FFT, corresponding to the pixels of the Spectrogram image. Regarding the architecture, summarized in Table 5, it was composed of 1D convolutional layers followed by three dense layers.

## 4. Results and Discussion

### 4.1. Evaluation Metrics

As evaluation metrics, the accuracy and F_1_ score were adopted, the latter defined as the harmonic mean of the Precision and Recall:

(11) $$F_{1}=2\times \frac{Precision\times Recall}{Precision+Recall},$$

Precision is the ratio between the true positives and all the positives,

(12) $$Precision=\frac{TP}{TP+FP},$$

and recall is the measure of how a model correctly identifies true positives, and it is mathematically identified as:

(13) $$Recall=\frac{TP}{TP+FN\;}.$$

In the end, the accuracy is defined as:

(14) $$Accuracy=\frac{TP+TN\;}{TP+FP+TN+FN},$$

where TP and TN are the True Positive and True Negative, and FP and FN are the False Positive and False Negative, respectively.

Finally, in the NN analysis, from the training report generated with Edge Impulse, both the estimated memory usage and the expected inference time were considered.

### 4.2. Performance Analysis

The models proposed in Section 3.3 and Section 3.4 were validated using the prototype described in Section 3.1. A custom-made waveform generator based on another NUCLEO F401RE was used to validate the system for each of the labels. At the end of each subsection, the performance of each of the adopted methods was compared against the relevant literature.

#### 4.2.1. Method 1: Performance Analysis

From the final training report, the NN achieved an accuracy of 99.4% in the final training and 89.40% in testing. According to the report generated with Edge Impulse, the expected memory RAM occupation was 2.1 kB, while for Flash, it was 65 kB, with an estimated inference time of 8 ms for ARM CORTEX M4 architecture. In terms of network optimization through the quantization technique, it is well-established that compromising accuracy can lead to more efficient memory usage and shorter inference times, making the network well-suited for real-time processing on microcontrollers. However, within this specific context, a significant degradation of NN functionality in terms of accuracy was not found. Instead, an improvement in the expected inference time and peak memory usage was observed. Specifically, the unoptimized NN featured 31 ms of expected inference time and 3.1 kB and 181.5 kB of RAM and Flash memory, respectively. The confusion matrix is shown in Table 6.

Multiple training phases were conducted to investigate the feasibility of an NN model based on statistical parameters. Throughout the training phase, two key considerations emerged regarding normalization and optimization. The training results are briefly presented in Table 7 and Table 8. Specifically, two approaches to data normalization were implemented: one based on Equation (15) and the other on (16). With congruent NN architecture, applying normalization based on (16) yielded a superior performance in terms of accuracy and the F1 score, resulting in a more balanced confusion matrix.

(15) $$y=\frac{x-min(x)}{\max\left(x\right)-min(x)}$$

(16) $$y=\frac{x}{max(abs(x))}$$

Developing firmware based on the flowchart in Figure 2, the system with the highest accuracy was validated using the prototype shown in Figure 1 and a custom-built waveform generator. Additionally, the processing times of the ECG signal and the time required for the NN inference were measured using a logic state analyzer. The NN performance was verified, executing inferences every 6 s and computing FIBFs and features every 500 ms, following the algorithm delineated in the block diagram in Figure 5. Finally, the inference time was measured to be 14.92 ms.

This approach optimized the methodology outlined in [14] to render it suitable for a microcontroller-based electronic device. The reduction in accuracy, specifically from 99.65 ± 0.23% to 89.40%, delineated a trade-off between energy consumption and model performance. In future works, consideration should be given to increasing the number of FIBFs, anticipating an accompanying increase in both memory usage and accuracy. However, this could entail the selection of a microcontroller with enhanced capabilities, thereby leading to higher energy dissipation.

#### 4.2.2. Method 2: Performance Analysis

The NN achieved an accuracy of 98.8% during final training and 94.76% during testing. According to the report generated with Edge Impulse, the expected RAM memory occupancy was 100.9 kB, while for Flash it was 514.7 kB, with an estimated inference time of 888 ms. To improve the performance of the neural network, it was optimized through quantization, achieving an accuracy of 99.3%, but with an expected inference time of 180 ms. Memory management also significantly improved, with peak RAM usage of 33.3 kB and Flash usage of 164.7 kB. Quantization played a key role in the optimization. Despite the lower precision, the accuracy of the model increased, demonstrating the effectiveness of this technique. In terms of inference time, there was a significant improvement, reducing from 888 to 180 ms. The overall optimization resulted in a more efficient NN that carefully balanced accuracy, inference time, and memory usage, making it suitable for implementation on resource-limited systems, such as microcontrollers or embedded systems.

The confusion matrix of the test phase is shown in Table 9.

**Figure 7:**
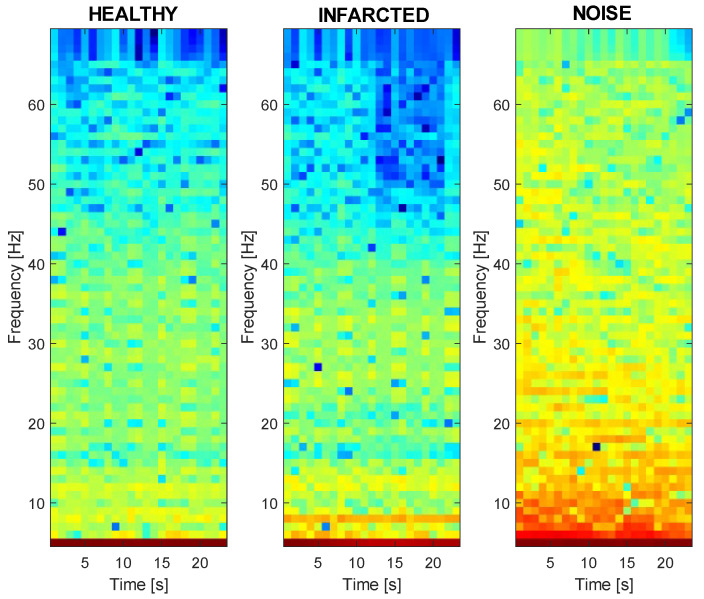
Spectrogram results for each label. Dark blue pixels corresponding to low amplitudes and brighter colors progressing up to red, indicating progressively stronger amplitudes.

In the design of this network, it was noticed that the major factors influencing training were the Spectrogram parameters and the optimal number of features. Much attention was paid to the Spectrogram parameter. The time interval was chosen to provide adequate time to identify at least one complete P-QRS-T. Regarding the noise floor, useful for normalization, it was set to −100 dB. Figure 7 shows the Spectrogram results for a sample of each label.

Table 10 shows the accuracy and F1 score values based on the number of features inputted into the NN. The final training involved 1495 input features.

Moreover, during the training process, various options were explored regarding activation functions and pooling techniques. Activation functions, including ReLU, LeakyReLU, Softmax, and Swish were considered. In the final training, Swish and Average Pooling were chosen as they yielded better results. The second approach was also validated on the same hardware. The NN operated correctly, with the NN pulse being invoked every 6 s, taking 830 ms for Spectrogram calculation and inference, in line with the algorithm outlined in the block diagram of Figure 6.

The literature contributions that adopted a comparable approach to Method 2 were those using II-lead ECGs, i.e., [24,27,30]. Limited studies in the literature have utilized experiments based on II-lead ECGs, with detailed performance comparisons illustrated in Table 11. Although exhibiting a slightly lower accuracy (94.76%) compared to the solution delineated in reference [27] (95.22%), this work approach distinguished itself by minimizing computational complexity, rendering it suitable for resource-constrained wearable devices. In contrast, reference [24] outperformed our method in wearable device memory occupancy, requiring only 20 kB, even if there was a diminished accuracy (84.36%) in comparison to this study’s solution. Lastly, compared to the study presented in [30], which employed a deep LSTM network, our work demonstrated superior performance in terms of accuracy (84.17%) and feasibility for wearable devices in real-time MI detection.

## 5. Conclusions

This paper discusses a study on different approaches based on the Edge Computing paradigm for MI detection. Two approaches were proposed for this purpose: one based on ML using FIBFs and another on DL using Spectrograms. For the ML approach, various normalization techniques were evaluated, including maximum value and range normalization. Instead, for the DL approach, the use of Spectrograms was analyzed, considering various parameters and their impact on training. During training, optimization was also performed to improve the performance of the NN using ARM Cortex-M4 architecture. Optimization techniques such as quantization were crucial in this context. The two models were compared in terms of accuracy, F1 score, expected inference times, and expected RAM and Flash memory usage, as indicated by the training report generated with Edge Impulse. Despite the lower inference time of the ML-based method (14.92 ms), the DL-based approach gave higher accuracy (94.76%) within a reasonable time frame (830 ms).

Validation was carried out using a prototype equipped with a 32-bit microcontroller, an AFE (Analog Front End) for ECG sensing, and a display for reading the NN inference results. The designed NNs successfully operated on the microcontroller, which not only acquired and processed data but also performed the NN inference.

## Figures and Tables

**Table 1:** Parameters employed in the final training of the first investigated method.

Parameter	Value
Learning rate	${8\times 10}^{-4}$
Number of epochs	250
Validation set size	20%
Number of features	240

**Table 2:** NN architecture for the first proposed method.

Layers	Parameters	F. Activation
Dense	128	swish
Dropout	0.3	
Dense	80	swish
Dropout	0.2	

**Table 3:** Parameters employed in the Spectrogram.

Parameter	Value
Frame length	1.4
Frame stride	0.2
FFT length	128
Noise floor (dB)	−100

**Table 4:** Parameters employed in the final training of the second investigated method.

Parameter	Value
Learning rate	${4\times 10}^{-4}$
Number of epochs	400
Validation set size	20%
Number of features	1495

**Table 5:** Parameters of each layer of the proposed NN.

Layers	Parameters	F. Activation
Reshape	input length/23	
Conv1D	128 kernel size = 5 padding = same	swish
AveragePooling1D	pool size = 2 strides = 2 padding = same	
Dropout	0.4	
Conv1D	64 kernel size = 5 padding = same	swish
AveragePooling1D	pool size = 2 strides = 2 padding = same	
Dropout	0.3	
Flatten		
Dense	60	swish
Dropout	0.3	
Dense	30	swish
Dropout	0.2	
Dense	20	swish

**Table 6:** Confusion matrix.

	Healthy	Infarcted	Noise	Uncertain
Healthy	85.1%	14.3%	0%	0.6%
Infarcted	11.9%	86.8%	0.3%	1.0%
Noise	0%	0%	100%	0%
F1 Score	0.86	0.87	1	

**Table 7:** Training report using (15).

Features	Accuracy	F1 Score
240	88.51	0.87 Infarcted 0.85 Healthy 0.97 Noise
260	87.05	0.87 Infarcted 0.83 Healthy 0.95 Noise
280	85.48	0.85 Infarcted 0.82 Healthy 0.96 Noise

**Table 8:** Training report using (16).

Features	Accuracy	F1 Score
220	87.08	Infarcted 0.87 Healthy 0.84 Noise 0.95
260	88.56	Infarcted 0.88 Healthy 0.85 Noise 0.98
280	87.40	Infarcted 0.86 Healthy 0.85 Noise 0.97

**Table 9:** Confusion matrix.

	Healthy	Infarcted	Noise	Uncertain
Healthy	91.8%	7.5%	0%	0.6%
Infarcted	2.4%	95.1%	1.4%	1.1%
Noise	0.2%	0.2%	99.4%	0.2%
F1 Score	0.94	0.94	0.98	

**Table 10:** Training report on the second investigated method.

Features	Accuracy	F1 Score
1105	91.72	Infarcted 0.90 Healthy 0.88 Noise 0.99
1365	92.73	Infarcted 0.92 Healthy 0.90 Noise 0.99
3315	91.95	Infarcted 0.91 Healthy 0.89 Noise 1.00

**Table 11:** Performance comparison of related works.

Related Works	Database	Lead	Accuracy (%)	Suitable for Wearable Device?
[27]	PTB	II	95.22	No
[24]	PTB-XL	II	84.36	Yes
[30]	PTB-XL	II	84.17	No
This work	Built Dataset	II	94.76	Yes

## Data Availability

The raw data supporting the conclusions of this article will be made available by the authors on request.
